# Genome Sequencing of *Paecilomyces Penicillatus* Provides Insights into Its Phylogenetic Placement and Mycoparasitism Mechanisms on Morel Mushrooms

**DOI:** 10.3390/pathogens9100834

**Published:** 2020-10-13

**Authors:** Xinxin Wang, Jingyu Peng, Lei Sun, Gregory Bonito, Yuxiu Guo, Yu Li, Yongping Fu

**Affiliations:** 1 Department of Plant Protection, Shenyang Agricultural University, Shenyang 110866, China; wangx220@msu.edu; 2 Engineering Research Center of Edible and Medicinal Fungi, Ministry of Education, Jilin Agricultural University, Changchun 130118, China; sunlei@jlau.edu.cn (L.S.); liyu@jlau.edu.cn (Y.L.); 3 Department of Plant, Soil, and Microbial Sciences, Michigan State University, East Lansing, MI 48842, USA; pengjin2@msu.edu (J.P.); bonito@msu.edu (G.B.); 4 Life Science College, Northeast Normal University, Changchun 130118, China; guoyx022@nenu.edu.cn

**Keywords:** *paecilomyces penicillatus*, whole genome sequencing, PacBio, phylogeny, carbohydrate-active enzymes (CAZymes)

## Abstract

Morels (*Morchella* spp.) are popular edible fungi with significant economic and scientific value. However, white mold disease, caused by *Paecilomyces penicillatus*, can reduce morel yield by up to 80% in the main cultivation area in China. *Paecilomyces* is a polyphyletic genus and the exact phylogenetic placement of *P. penicillatus* is currently still unclear. Here, we obtained the first high-quality genome sequence of *P. penicillatus* generated through the single-molecule real-time (SMRT) sequencing platform. The assembled draft genome of *P. penicillatus* was 40.2 Mb, had an N50 value of 2.6 Mb and encoded 9454 genes. Phylogenetic analysis of single-copy orthologous genes revealed that *P. penicillatus* is in Hypocreales and closely related to Hypocreaceae, which includes several genera exhibiting a mycoparasitic lifestyle. CAZymes analysis demonstrated that *P. penicillatus* encodes a large number of fungal cell wall degradation enzymes. We identified many gene clusters involved in the production of secondary metabolites known to exhibit antifungal, antibacterial, or insecticidal activities. We further demonstrated through dual culture assays that *P. penicillatus* secretes certain soluble compounds that are inhibitory to the mycelial growth of *Morchella sextelata*. This study provides insights into the correct phylogenetic placement of *P. penicillatus* and the molecular mechanisms that underlie *P. penicillatus* pathogenesis.

## 1. Introduction

Morels (*Morchella* spp.) are valuable edible fungi that belong within the order Pezizales. There is a long history of morel consumption in Asia, Europe, and North America [1]. The increasing demand for dried and fresh morels has driven the growth of the industry and the expansion of suitable morel cultivation areas [2,3]. In China, the morel cultivation area reached approximately 9000 ha in the production season of 2018–2019 and continues to expand [4]. With the expanding cultivation range and density of production, disease has become a major limiting factor for morel production. Typical diseases include stipe rot disease caused by the *Fusarium incarnatum*—*F. equiseti* species complex and pileus rot disease caused by *Diploöspora longispora* [5,6]. White mold disease, caused by *Paecilomyces penicillatus*, is another major disease affecting morels. This disease was first reported in Sichuan, China in 2013 [7]. *P. penicillatus* infection in one location can quickly spread to neighboring morel cultivation areas and can account for up to 80% of morel yield losses. Currently, no commercial fungicide has been registered to control for morel white mold disease, and the management of this disease relies primarily on the removal of the symptomatic fruiting bodies. The molecular basis of *P. penicillatus* pathogenicity is, to the best of our knowledge, unknown. This makes it difficult to develop tailored solutions to control this disease.

*Paecilomyces* is a polyphyletic genus, and *Paecilomyces* spp. are classified within Sordariomycetes or Eurotiomycetes based on phylogenetic analyses of 18S rRNA sequences, internal transcribed spacer (ITS) sequences and 5.8S rDNA sequences [8,9]. *P. penicillatus* was first isolated from rotten mushrooms by Samson in 1974 and was originally placed in Trichocomaceae, order Eurotiales, class Eurotiomycetes based on the morphological characteristics of this pathogen [10]. However, molecular phylogenetic analyses based on β-tubulin and 18S rRNA sequences later placed *P. penicillatus* within the order Hypocreales of Sordariomycetes, and revealed an uncertain affinity with Clavicipitaceae [8,11]. In addition to Clavicipitaceae, Hypocreales also contains seven other families, namely Hypocreaceae, Bionectriaceae, Cordycipitaceae, Nectriaceae, Niessliaceae, Ophiocordycipitaceae, and Stachybotryaceae. With the rapid development of sequencing technologies, an increasing number of fungal genomes have become available [12,13,14]. Phylogenetic analysis of the single-copy orthologous genes has been widely used for evolutionary studies and provides improved resolution for elucidating the phylogenetic relationships of organisms compared to the use of sequences from one or a few genes [13,15,16,17]. Due to the lack of *P. penicillatus* genomic resources, a comprehensive phylogenetic analysis based on single-copy orthologous genes is not available.

The molecular mechanisms of mycoparasitism in *P. penicillatus* remain unclear. Though not known from *P. penicillatus*, lysis of the host cell wall is a common mechanism of mycoparasitism [18]. Many mycoparasitic fungi have been identified in Hypocreaceae, including *Cladobotryum dendroides* and *Cladobotryum protrusum*, which can cause cobweb disease, as well as *Hypomyces perniciosus*, the causative agent of wet bubble disease [13,15,16]. In particular, *Trichoderma* spp. have been used as model systems to study the mechanisms of mycoparasitism [19]. For instance, *Trichoderma harzianum*, the causative agent of green mold disease on white button mushroom, produces various fungal cell wall-degradation enzymes that are being used in biotechnology and in biological control [20,21,22]. Genome analysis of *Trichoderma atroviride* and *Trichoderma virens* indicated that they contain a large numbers of genes encoding fungal cell wall-degradation enzymes compared to non-mycoparasitic fungi [18].

In this study, we obtained the first genome sequence of *P. penicillatus* using the single-molecule real-time (SMRT) sequencing platform [23]. Through phylogenetic analysis of single-copy orthologous genes, we determined the phylogenetic placement of *P. penicillatus*. Based on carbohydrate-active enzymes (CAZymes) analysis, secondary metabolite analysis, and virulence factor analysis, we demonstrated the putative molecular mechanisms of mycoparasitism of *P. penicillatus*. This study and the genomic resources made available herein provide a basis for the future functional characterization of the pathogenesis-related genes in *P. penicillatus* as well as the development of morel white mold disease management strategies.

## 2. Materials and Methods

### 2.1. Fungal Material

The *P. penicillatus* strain CCMJ2836 was provided by the Engineering Research Center of the Chinese Ministry of Education for Edible and Medicinal Fungi, Jilin Agricultural University (Changchun, China), which was isolated from white mold-diseased morel fruiting bodies in Sichuan Province, China. The mycelial plugs of strain CCMJ2836 were grown on potato glucose agar (PDA) medium overlaid with cellophane sheets for 10 d at 25 °C under a light/dark photoperiod (12/12 h).

### 2.2. Genome Sequencing and Assembly

*P. penicillatus* CCMJ2836 genomic DNA was extracted from the mycelia using NuClean Plant Genomic DNA Kits (CWBIO, Beijing, China) following the manufacturer’s instructions. The 20 kb-gDNA library was prepared and sequenced on two SMRT cells with a PacBio Sequel system. Raw data were de novo assembled via SMARTdenovo (https://github.com/ruanjue/smartdenovo). Genome assembly completeness was assessed with both the Core Eukaryotic Genes Mapping Approach (CEGMA) [24] and Benchmarking Universal Single-Copy Orthologs (BUSCO; [25]). Protein-encoding genes were predicted with GLEAN [26] (http://sourceforge.net/projects/glean-gene) through both extrinsic and ab initio algorirhms.

### 2.3. Gene Prediction and Genome Annotation

Annotations of the predicted genes were obtained through BLASTp (*e*-value ≤ 1 × 10^−5^) referencing the following databases: InterPro, TrEMBL, National Center for Biotechnology Information non-redundant (NCBI-nr), Swiss-Prot [27], Gene Ontology (GO) [28], Clusters of Orthologous Groups (COG) [29], and Kyoto Encyclopedia of Genes and Genomes (KEGG) [30]. Transposon elements and tandem repeat sequences (TRF) were predicted with RepeatMasker (http://www.repeatmasker.org) and Tandem Repeat Finder [31], respectively. Transfer RNAs (tRNAs) were predicted with tRNAscan-SE [32]. Ribosomal RNA (rRNA) sequences were identified with rRNAmmer [33] following both evidence-based and ab initio approaches. Non-coding small RNAs were annotated with the Rfam database [34].

### 2.4. Phylogenetic Analysis of Single-Copy Orthologs

Reciprocal BLASTp searches were conducted (*e*-value ≤ 1 × 10^−7^) with protein sequences from the representative species of Eurotiomycetes (four Eurotiales species: *Paecilomyces variotii* [35], *Penicillium chrysogenum* [36], *Aspergillus nidulans* [37], and *A. niger* [38]) and Sordariomycetes (Hypocreales species: *Metarhizium robertsii* [39], *T. atroviride* [40], *T. harzianum* [41], *Trichoderma reesei* [42], *T. virens* [43], *C. dendroides* [16], *C. protrusum* [13], *H. perniciosus* [15], *Cordyceps militaris* [44], *Fusarium graminearum* [45], and *Niesslia exilis* (https://mycocosm.jgi.doe.gov/Nieex1), *Ophiocordyceps unilateralis* [46], *Stachybotrys chartarum* [47], and one Sordariales species: *Neurospora crassa* [48]) were included to obtain single-copy ortholog sequences. *Schizosaccharomyces pombe* [49] in Schizosaccharomycetes was used as an outgroup for this analysis. Alignments with high-score pairs (alignment ratio ≥ 30%) were conjoined by SOLAR (V0.0.19) and used for the subsequent phylogenetic analysis. Sequences of single-copy orthologs were aligned with MUSCLE [50], and the phylogenetic relationships of the species were estimated using the maximum likelihood (ML) model in RA × ML [51] with 1000 bootstrap replicates. *Schizosaccharomyces pombe* was included in this analysis as an outgroup taxon.

### 2.5. Carbohydrate-Active Enzyme (CAZyme) Family Analysis

The deduced amino acid sequences of the *P. penicillatus* genes were searched against the CAZy database (http://www.cazy.org/) [52] with BLASTp (*e*-value ≤ 1 × 10^−5^, identity ≥ 40% and coverage ≥ 40%), and were further grouped into auxiliary activities (AAs), carbohydrate-binding modules (CBMs), carbohydrate esterases (CEs), glycoside hydrolases (GHs), glycosyl transferases (GTs), and polysaccharide lyases (PLs). Modular analysis of GH family 18 was carried out with InterProScan (http://www.ebi.ac.uk/Tools/InterProScan/). Signal peptides of GH family 18 were predicted using the SignalP 5.0 server (http://www.cbs.dtu.dk/services/SignalP-5.0/).

### 2.6. Secondary Metabolite Gene Cluster Analysis and Pathogenicity-Related Genes

Gene clusters encoding secondary metabolite enzymes were analyzed with AntiSMASH fungal version 3.0 (https://fungismash.secondarymetabolites.org/) with the following built-in features activated: ActiveSiteFinder, ClusterBlast, Cluster Pfam analysis, KnownClusterBlast, Pfam-based GO term annotation, and SubClusterBlast. The cutoff similarity value for a known cluster was 40%. Genes within the clusters were further searched against the Pathogen-Host Interactions database (PHI-base) and the database of fungal virulence factor (DFVF) by BLASTp (*e*-value ≤ 1 × 10^−5^, identity ≥ 40% and coverage ≥ 40%) to predict the putative involvement of the gene clusters in the pathogenesis of *P. penicillatus.* Pathogenic genes were identified using the PHI (http://www.phi-base.org/) [53] and DFVF (http://sysbio.unl.edu/DFVF/Download.php) [54] databases.

### 2.7. Dual Culture Assay

Cultures of *P. penicillatus* strain CCMJ2836 and *M. sextelata* strain GB-Ch-102 were inoculated on HIMEDIA corn meal peptone yeast agar (CMA) and incubated at room temperature for 5 d. *P. penicillatus* agar discs were inoculated on one side of regular or two-compartment CMA dishes. After 6 d, *M. sextelata* agar discs were inoculated on the other side of the petri dish at about 4 cm from the *P. penicillatus* agar disc and then incubated for two more days. Each confrontation culture was replicated 10 times and incubated at room temperature. Cultures were photographed with an Epson Perfection V700 Photo scanner.

## 3. Results and Discussion

### 3.1. Genome Sequencing and Assembly of P. penicillatus

The genome of *P. penicillatus* CCMJ2836 strain was sequenced on the PacBio SMRT Sequel platform using two SMRT cells. A total of 4,266 Mb clean data (~105 × coverage) was generated (Table S1). The de novo assembly of the *P. penicillatus* genome was ~40.20 Mb, consisting of 52 scaffolds with 2.60 Mb in N50 value and 44.70% in guanine-cytosine (GC) content (Table 1). The completeness of the *P. penicillatus* genome was assessed through the CEGMA and BUSCOs analyses, with completeness scores of 95.56% and 97.20%, respectively. The whole genomic sequence of *P. penicillatus* CCMJ2836 has been deposited at GenBank and is available under accession number JACGSR000000000. The version described in this paper is version JACGSR010000000. 

### 3.2. Gene Prediction and Annotation

Combining both homology-based and ab initio gene prediction approaches, 9454 protein-coding genes were predicted in *P. penicillatus*, with an average sequence length of 1694.45 bp. On average, each gene contains 2.61 exons in 557.93 bp. The putative protein-coding genes were functionally annotated using the following databases: NCBI nr (95.75%), Swiss-Prot (63.48%), KEGG (62.07%), COG (44.16%), TrEMBL (95.65%), InterPro (70.85%), and GO (49.55%). Overall, 96.34% genes had homologs in at least one database (Table S2 and Text S1). We identified ~3.1 Mbp repeat sequences and 0.12% non-coding RNA species (ncRNA) in the *P. penicillatus* genome. Among these repeat sequences, long terminal repeats (LTRs) were the most abundant representing 5.64% of the genome (Table S3 and Text S1). The ncRNA species contained 197 transfer RNA (tRNA) genes, 87 microRNA (miRNA) genes, 38 ribosomal RNA (rRNA) genes, and 25 small nuclear RNA (snRNA) genes (Table S4 and Text S1).

### 3.3. Phylogenomic Analysis

**Figure 1:**
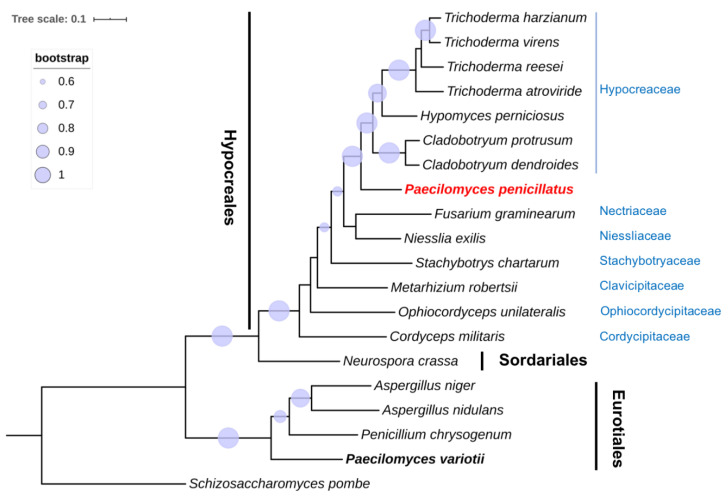
Phylogenetic analysis of single-copy orthologous genes of *P. penicillatus* and 13 representative species of Hypocreales, one Sordariales species, and four Eurotiales species. *S. pombe* was used as an outgroup for this analysis. *P. variotii* is indicated in bold font, and *P. pencililatus* is highlighted in red font. Families of Hypocreales are labeled in blue font. The phylogenetic tree was generated using the maximum-likelihood method with 1000 bootstrap replicates. The tree scale indicates residue substitutions per site. Only bootstrap values greater than 0.6 are displayed.

In view of the polyphyletic status of the *Paecilomyces* genus and the uncertain phylogenetic placement of *P. penicillatus*, a phylogenetic analysis was conducted using the single-copy orthologous genes (Figure 1). As expected, species in Eurotiomycetes and Sordariomycetes were clearly separated into two phylogenetic clades. In agreement with the conclusion made from both morphological characteristics and ITS sequences [9], *P. variotii* was placed under Eurotiomycetes. Within Sordariomycetes, Hypocreales species grouped together and were separated from *N. crassa* in Sordariales. *P. penicillatus* was placed under Hypocreales and was closest to Hypocreaceae, which includes many mycoparasitic fungi that can infect edible mushrooms. The next closest families were Nectriaceae and Niessliaceae. *P. penicillatus* is distantly related to Stachybotryaceae, Clavicipitaceae and Ophiocordycipitaceae, and has the most distant relationship with Cordycipitaceae. Our results indicate that *Paecilomyces* is polyphyletic and that *P. penicillatus* and *P. variotii* are clearly distantly related. We conclude that *P. penicillatus* is in the order of Hypocreales but not Eurotiales, which was previously concluded based on morphological characters. Our findings support a previous study based on 18S rRNA and β-tubulin gene sequences [8,11]. Our phylogenetic analysis further demonstrated that *P. penicillatus* is closely related to *Hypocreaceae*. A previous phylogenetic analysis of the 5.8S rDNA and ITS sequences of entomopathogenic *Paecilomyces* spp. demonstrated that most *Paecilomyces* species are Hypocreales and a few *Paecilomyces* species are Eurotiales. Within Hypocreales, *Paecilomyces* spp. was further classified into three subgroups. One subgroup includes *Paecilomyces viridis*, *P. penicillatus*, and *Paecilomyces carneus*. Most strains of *Paecilomyces lilacinus* and *Paecilomyces marquandii* are closely related and are classified as a distinct subgroup. The other subgroup contains *Paecilomyces farinosus*, *Paecilomyces fumosoroseus*, and other *Paecilomyces* species [9]. Due to the lack of genomic resources of most *Paecilomyces* spp., a single-copy orthologous genes-based phylogenetic analysis is not technically possible yet. Our study, therefore, lays the foundation for further phylogenetic classification of *Paecilomyces* spp. by deploying the single-copy orthologous gene sequences.

### 3.4. The CAZyme

**Figure 2:**
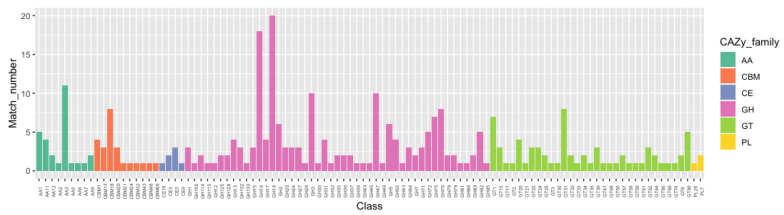
Annotation of carbohydrate-related genes in the *P. penicillatus* genome.

To successfully infect fungal hosts, mycoparasitic fungi commonly produce chitinases and glucanases to aid in the digestion of the fungal host cell wall, which is composed of chitin and glucan [19,55,56]. In light of the mycoparasitic nature of *P. penicillatus*, we hypothesized that *P. penicillatus* may also produce enzymes that can degrade the cell wall of its host. To assess this possibility, the genome of *P. penicillatus* was mapped to the CAZymes database (http://www.cazy.org), which includes all the enzyme families known to function in carbohydrate metabolism [57]. A total of 299 CAZymes were identified including 170 GHs, 67 GTs, 28 AAs, 24 CBMs, seven CEs, and three PLs (Figure 2). The most abundant CAZyme family in *P. penicillatus* is the GH family 18. The second and the third most abundant CAZyme families are the GH family 16 and the AA family 3, respectively (Figure 2).

**Figure 3:**
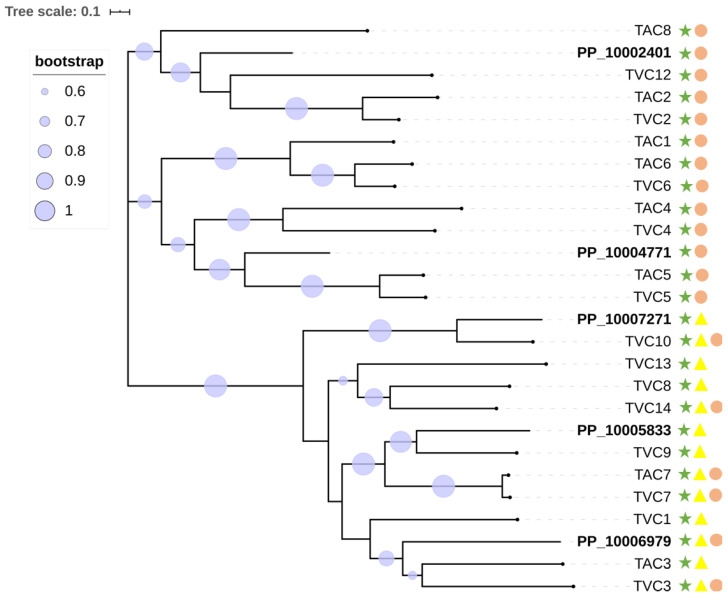
Phylogenetic relationships of *P. penicillatus*, *T. atroviride,* and *T. virens* sgC chitinases. The presence of GH18 modules (IPR001223) is indicated with green stars, LysMs (IPR018392) are indicated in yellow triangles, and CBM18s (IPR001002) are represented by pink circles.

The GH family 18 contains all the enzymes that have been previously shown to contribute to the degradation and replenishment of both intrinsic and extrinsic fungal chitin [58] (Figure 2). GH family 18 can be further divided into three subgroups, namely A, B, and C [59,60]. We were particularly interested in subgroup C (sgC) chitinases due to their potential involvement in mycoparasitism and their other interesting features [41,61]. Currently, known sgC chitinases contain CBM 18 (chitin binding) domains and/or CBM 50 (LysM) domains [18,62]. An analysis of sgC chitinases was performed. Based on the domain prediction, out of the 20 chitinases in GH family 18, five were predicted to be in sgC. Three of them have the CBM 18 domain and the other two have both the CBM 18 and the CBM 50 domains. In view of the close phylogenetic relationship between *P. penicillatus* and *Trichoderma* spp. and the well-studied production of fungal cell wall-degradation enzymes by *Trichoderma* spp., a phylogenetic analysis of sgC chitinases of *P. penicillatus, Trichoderma atroviride* and *T. virens* was conducted (Figure 3). Interestingly, our results showed that out of five genes, only two orthologue pairs could be found in the sgC chitinases of *P. penicillatus* and *T. virens* (PP_10005833/TVC9, PP_10007271/TVC10). The drastic differences in the number and sequence divergence of sgC in these phylogenetically closely-related mycoparasitic organisms suggests a strong evolutionary pressure on this protein family.

Beside chitinases, β-(1,3)-glucanases are another enzyme family that decomposes the fungal cell wall and belongs to GH family17, 55, 64, and 81 [41]. Compared with *Trichoderma* spp. and other representative fungal pathogens of plants or animals, *P. penicillatus* and *Trichoderma* spp. encode the highest number of GH family 18 proteins among the fungal organisms included in this study [41]. For GH families 64 and 81, we found a higher number in *P. penicillatus* and *Trichoderma* spp. in comparison to the other filamentous fungi. Similarly, *P. penicillatus* contains the highest number of GH family 75, which could be involved in the decomposition of chitinous carbohydrates [41]. Our results indicated that *P. penicillatus* produces a plethora of chitinases and β-(1,3)-glucanases in resemblance to the well-characterized mycoparasitic *Trichoderma* spp.

### 3.5. Secondary Metabolites and Pathogenicity-Related Genes

We identified 74 putative secondary metabolite biosynthesis gene clusters in *P. penicillatus*. Among them, seven gene clusters had significant hits to known secondary metabolite biosynthesis gene clusters (Table 2), including two antifungal (squalestatin 1 and AbT1), one antibacterial (cephalosporin C), one insecticidal (leucinostatin), two mammal-toxic (monascorubrin and 6-methylsalicyclic acid), and one plant-toxic (dimethylcoproge) secondary metabolic compounds. Multiple *P. penicillatus* genes in these gene clusters showed significant sequence similarity to genes in the Pathogen-Host Interactions database (PHI-base) and/or the database of fungal virulence factor (DFVF), indicative of possible involvement of these gene clusters in the pathogenesis of *P. penicillatus*. Specifically, Squalestatin 1 (40% similarity) is a potent inhibitor of squalene synthase, an essential enzyme for sterol biosynthesis [63]. Squalestatin 1 exhibits broad-spectrum antifungal activity [64] and was identified in *Phoma* sp. and several ascomycetes [65,66]. AbT1 (100% similarity) is a precursor of the cyclic peptide antibiotic Aureobasidin A (AbA), known to be produced by *Aureobasidium pullulans* [67]. AbA has been shown to exhibit inhibitory activity to yeast inositol phosphorylceramide synthase and is toxic to several other fungal organisms, such as *Aspergillus nidulans* and *A. niger* [67,68]. The genome of *P. penicillatus* also contains a gene cluster that has a 40% similarity to that of a gene involved in the biosynthesis of leucinostatin, a compound with antitrypanosomal properties. Interestingly, most *Paecilomyces* spp. identified thus far are entomophagous [69], and leucinostatin was previously reported to be produced by a *Paecilomyces* sp. isolated from soil [70]. In addition, several causative gene clusters for mammal or plant mycotoxins or their precursors were also predicted in *P. penicillatus* with 100% similarity, including dimethylcoproge [71], monascorubrin [72], and 6-methylsalicyclic acid [73]. It remains to be investigated whether these mycotoxic secondary compounds facilitate the infection of *P. penicillatus* on morel mycelia and fruiting bodies, or whether they coordinate or contribute to interspecies competition against other microbes living in the same environmental niche.

### 3.6. Dual Culture Assay

**Figure 4:**
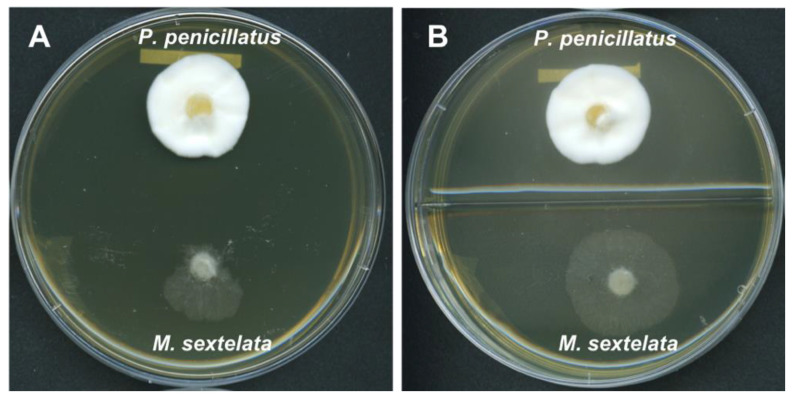
Confrontation cultures between *P. penicillatus* and *M. sextelata* on CMA medium. (**A**). Normal petri dish (**B**). Two-compartment petri dish.

Based on the above analyses, we hypothesized that *P. penicillatus* may produce certain inhibitory compounds that can affect the hyphal growth of morels. To test this possibility, we conducted dual culture assays where agar plugs of both *P. penicillatus* and *M. sextelata* were inoculated onto the same petri dish. Indeed, *M. sextelata* had minimum growth in the direction towards the colony of *P. penicillatus*, and extensive growth of *M. sextelata* was observed in the direction away from *P. penicillatus* (Figure 4A), suggesting that *P. penicillatus* does produce host-inhibitory substances in a contact-independent manner. In an attempt to further investigate whether the substances are volatile or soluble, we also conducted confrontation assays on enclosed two-compartment petri dishes, where *P. penicillatus* and *M. sextelata* were inoculated on the center of each compartments. We expected to observe a similar inhibitory effect of *P. penicillatus* on *M. sextelata* in the case of volatile compounds being produced by *P. penicillatus.* However, *M. sextelata* grew rapidly in all directions at nearly equal speed in these assays (Figure 4B). This indicated that the inhibitory compounds were unlikely to be volatile, and rather, that they must be secreted and diffused in the medium. We are currently investigating whether *P. penicillatus* exhibits the antagonistic activity against *M. sextelata* through extracellular fungal cell wall-degradation enzymes or mycotoxins, as predicted in this study.

## 4. Conclusions

In this study, we present the first whole-genome sequence of *P. penicillatus*, the causative agent of the morel white mold disease. The genome of *P. penicillatus* is ~40.20 Mb in size with an N50 value of 2.59 Mbp and 9454 coding genes. The phylogenetic analysis based on the single-copy orthologous genes demonstrated that *P. penicillatus* is within Hypocreales and is closely related to Hypocreaceae, a family that is known to include many mycoparasitic species. CAZymes analysis further illustrated that the genome of *P. penicillatus* has a large number of fungal cell wall degradation enzymes. We identified a plethora of gene clusters that are known to be involved in the biosynthesis of cytotoxic secondary compounds that may function coordinatively during the pathogenesis of *P. penicillatus*. We also showed that *P. penicillatus* can produce contact-independent soluble host-inhibitory compounds. Taken together, we provide strong evidence in support of the phylogenetic placement of *P. penicillatus* within Hypocreales and provide a basis for the future functional characterization of the fungal cell wall-degradation enzymes and secondary metabolites produced by this mycoparasitic fungus.

## Figures and Tables

**Table 1:** Summary of the *P. penicillatus* CCMJ2836 genome.

Genome Size (Mbp)	40.20
Contig number	52
Contig N50 (Mbp)	2.60
Contig N90 (Mbp)	0.33
GC content (%)	44.70
Annotated protein-coding genes	9454
Repeat sequences proportion (%)	7.65
ncRNA proportion (%)	0.12
CEGMA^1^ completeness score (%)	95.56
BUSCO^2^ completeness score (%)	97.20

^1^ Core Eukaryotic Genes Mapping Approach (CEGMA); ^2^ Benchmarking Universal Single-Copy Orthologs (BUSCO).

**Table 2:** Summary of secondary metabolite biosynthesis gene clusters in *P. penicillatus* with known functions and their putative involvement in pathogenesis.

Secondary Metabolite	Similarity (%)	Gene ID	PHI-Base *	DFVF **
Squalestatin S1	40	PP_10006022 - PP_10006026		+
Leucinostatin	40	PP_10000538 - PP_10000553	+	
Cephalosporin C	71	PP_10005054 - PP_10005084	+	
Dimethylcoprogen	100	PP_10006836 - PP_10006847	+	+
Monascorubrin	100	PP_10007343 - PP_10007359		+
6-methylsalicyclic acid	100	PP_10002195 - PP_10002216	+	+
AbT1	100	PP_10000461 - PP_10000471		+

* One or more genes within the cluster have significant hits in the Pathogen-Host Interactions database (PHI-base). ** One or more genes within the cluster have significant hits in the database of fungal virulence factor (DFVF).

## Supplementary Materials

The following are available online at https://www.mdpi.com/2076-0817/9/10/834/s1, Text S1: Annotation of the genome of *P. penicillatus*, Table S1: CEGMA analysis on the completion of the *P. penicillatus* genome, Table S2: Functional annotation of the genes in the *P. penicillatus* genome against several databases, Table S3: The statistics of the repeat sequences in the *P. penicillatus* genome, Table S4: Annotation of tRNA, rRNA, miRNA, and snRNA in the *P. penicillatus* genome.
